# Microsatellite instability and its associations with the clinicopathologic characteristics of diffuse large B‐cell lymphoma

**DOI:** 10.1002/cam4.2870

**Published:** 2020-02-05

**Authors:** Tian Tian, Jiwei Li, Tian Xue, Baohua Yu, Xiaoqiu Li, Xiaoyan Zhou

**Affiliations:** ^1^ Department of Pathology Fudan University Shanghai Cancer Center Shanghai China; ^2^ Department of Oncology Shanghai Medical College Fudan University Shanghai China; ^3^ Institute of Pathology Fudan University Shanghai China

**Keywords:** chemotherapy response, complete response, DLBCLs, MSI

## Abstract

Microsatellite instability (MSI) has been investigated as a prognostic and predictive factor for chemotherapy in colorectal cancer and has recently been demonstrated to be predictive of the PD‐1/PD‐L1 checkpoint blockade response in various solid tumors. However, MSI status in diffuse large B‐cell lymphomas (DLBCLs) has not been thoroughly explored. This study investigated MSI status in DLBCLs and analyzed the associations between MSI and clinicopathologic characteristics and clinical outcomes. Ninety‐two cases of primary DLBCLs treated with R‐CHOP/CHOP chemotherapy between 2009 and 2017 were collected. MSI detection was performed by the Promega MSI Analysis System. The protein expression of *MLH1*, *MSH2*, *MSH6*, and *PMS2* was detected by immunohistochemistry. The associations of MSI‐H and MSI‐L with progression‐free survival (PFS) and overall survival (OS) were assessed by COX models and Kaplan–Meier curves. The correlations of complete response (CR) after R‐CHOP/CHOP chemotherapy with MSI‐H and MSI‐L were examined by univariate and multivariate logistic regression analyses, respectively. 3 of 92 cases (3.2%) were high MSI (MSI‐H), and 9 cases (9/92, 9.8%) exhibited low MSI (MSI‐L). One case with MSI‐H showed negative expression of *MSH2* and *MSH6*. Univariate analysis indicated that MSI‐L was correlated with poor response to R‐CHOP/CHOP chemotherapy in DLBCLs (OR, 0.178; 95% CI, 0.041‐0.776; *P* = .022). Multivariate analysis showed that MSI‐L was an independent predictive factor for non‐CR to R‐CHOP/CHOP chemotherapy (OR, 0.144; 95% CI, 0.027‐0.761; *P* = .023). Kaplan‐Meier curves showed that there was a trend that MSI‐H patients had favorable PFS (*P* = .36) and OS (*P* = .48), which did not have statistical significance and MSI‐L was not significantly correlated with PFS (*P* = .24) and OS (*P* = .52).Our study indicated that there existed MSI‐H and MSI‐L in DLBCLs. MSI‐L could be an independent predictive factor for the chemotherapy response in DLBCLs.

## Graphical abstract

There existed MSI‐H and MSI‐L in DLBCLs. The MSI‐L phenotype could be an independent predictive factor to the poor response of R‐CHOP/CHOP chemotherapy in DLBCLs.

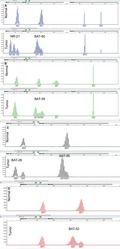

## 1 INTRODUCTION

Diffuse large B‐cell lymphoma (DLBCL) is the most common lymphoid malignancy in adults, accounting for nearly 35% of non‐Hodgkin lymphomas.^1^ Though DLBCL is curable with combination chemotherapy (rituximab, cyclophosphamide, doxorubicin, vincristine, and prednisolone, R‐CHOP) in up to 60% of patients, there also exist patients developing progressive disease.^2^ Therefore, discovering new predictive and prognostic factors and developing new targeted therapies is significant.

Microsatellite instability (MSI) implies the somatic destabilization of short reiterated motifs, which reflects defective DNA mismatch repair (dMMR). Mismatch repair (MMR) is a crucial DNA repair way that counteracts errors caused by DNA polymerases in the course of DNA replication. Fifteen percent of colorectal cancer patients have MSI, and dMMR has been demonstrated to be related with favorable outcomes and a predictive factor for lack of efficiency of 5‐fluorouracil (5‐FU) in colorectal cancer. It is now well known that dMMR caused tumor resistance against a series of antineoplastic agents.^3^, ^4^ Thus, MSI has been considered as a predictive candidate biomarker in the field of oncology.

Many cancers encode mutation‐associated neoantigens (MANAs), which might be potential determinants of immune checkpoint blockades, like PD‐1/PD‐L1 blockades.^5^, ^6^, ^7^ MMR‐deficient cancers have large numbers of MANAs which would stimulate the immune response.^8^ Recent studies have demonstrated that dMMR could predict the response to PD‐1 inhibitor in 12 different kinds of solid tumors, including cholangio carcinoma, endometrial cancer, neuroendocrine carcinoma, and osteosarcoma and so on.^9^ Therefore, the evaluation of MSI and MMR deficiency has significance in predicting the response to PD‐1/PD‐L1 checkpoint blockades in various types of cancers.

A recent study reported that 5 of 28 DLBCL cases had MSI in the cohort.^10^ However, the sample size of DLBCLs in that investigation was small. In addition, the MSI status and its associations with clinicopathologic features and clinical outcomes in DLBCLs are still largely unknown. Thus, in our study, we investigated the MSI status in DLBCLs and examined the correlations between MSI and clinicopathologic features and the clinical outcomes, including chemotherapy response and patient prognosis.

## 2 MATERIALS AND METHODS

### 2.1 Patients and specimens

**Figure 1:**
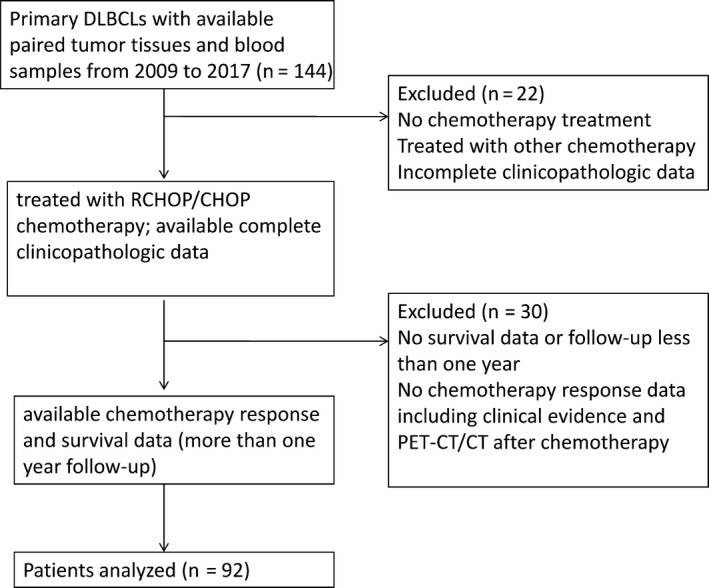
Flowchart of exclusion criteria of the cohort

A total of 92 cases of primary DLBCLs were derived from the pathology database of the Fudan University Shanghai Cancer Center between 2009 and 2017. All cases were diagnosed by two pathologists (Xue, Yu) according to the criteria of the World Health Organization (WHO) classification of hematopoietic and lymphoid tissues.^1^ The inclusion criteria included: primary DLBCLs, available paired tumor tissues and blood samples, treated with R‐CHOP/CHOP chemotherapy, available complete clinicopathologic data including age, sex, International Prognostic Index (IPI) scores, Ann Arbor stage, B symptoms, serum lactate dehydrogenase (LDH), and molecular type identified by immunohistochemistry (germinal center B‐cell like (GCB) vs non‐GCB) and available chemotherapy response data (evaluated by clinical evidence and PET‐CT/CT after chemotherapy) and survival data (more than 1 year's follow‐up). The flowchart of exclusion criteria is shown in Figure 1. The clinicopathologic data, including sex, age, International Prognostic Index (IPI) scores, Ann Arbor stage, B symptoms, serum lactate dehydrogenase (LDH), and molecular type (germinal center B‐cell like (GCB) vs non‐GCB) and survival data, were retrospectively reviewed. Median follow‐up was 41 months (range: 12‐116 months). Fresh frozen specimens of 92 cases and their matched blood samples were collected from the Tissue Bank at Fudan University Shanghai Cancer Center.

### 2.2 DNA extraction

DNA extraction from 92 cases of fresh frozen tissues and their matched blood samples was carried out using the QIAamp DNeasy Blood & Tissue Kit according to the manufacturer's protocol (Qiagen), respectively. DNA concentration was measured by a Nanodrop 2000 spectrophotometer (Thermo Fisher Scientific).

### 2.3 MSI

Microsatellite instability analysis was performed in 92 cases of DNA samples isolated from tumor tissues and 92 cases of DNA samples from matched blood samples, which were regarded as normal controls. MSI was detected by a Promega MSI Analysis System (Version 2.0) according to the manufacturer's protocol (Promega). The MSI Analysis System included fluorescently labeled primers for the coamplification of eight markers, including eight mononucleotide repeat markers (BAT‐25, BAT‐26, BAT‐52, BAT‐56, BAT‐59, BAT‐60, NR‐21, and MONO‐27) and two pentanucleotide repeat markers (Penta C and Penta D). The PCR products were separated byABI 3500DX Genetic Analyzer (Thermo Fisher Scientific).Then the output data were analyzed with GeneMapper^®^ Analysis Software (Thermo Fisher Scientific) to identify MSI status. The results were classified as high microsatellite instability (MSI‐H) (microsatellite alterations in at least 2 of the 8 markers), low microsatellite instability (MSI‐L) (microsatellite alteration in one marker), and microsatellite stable (MSS) (no microsatellite alteration) according to the National Cancer Institute (NCI) criteria and the manufacture's protocol (Promega).^11^

### 2.4 Immunohistochemistry

Immunohistochemistry were carried out on formalin‐fixed, paraffin‐embedded (FFPE) tissue sections of DLBCL with the MSI‐H or MSI‐L phenotypes using antibodies directed against MSH2, MSH6, MLH1, and PMS2 (Abcam, ab252190). It was recorded as negative expression when nuclear staining was absent from all tumor cells but preserved in stromal cells. The positive expression of MMR proteins was positive nuclear staining in more than 10% (≥10%) of tumor cell nuclei with positive staining of internal control cells. Staining in less than 10% of tumor cells was classified as indeterminate and equivocal as before described.^12^

### 2.5 Statistical analysis

The associations between the MSI status and clinicopathologic features were analyzed by Pearson's chi‐square test, Mann‐Whitney test or the Fisher's exact test. For the survival analyses, the endpoints were progression‐free survival (PFS) and overall survival (OS).^13^ OS was counted from the date of diagnosis to the date of death from any cause. PFS was counted from the date of diagnosis to the date of lymphoma recurrence or death from any cause or the date of last follow‐up. The lymphoma recurrence meant relapsed disease or progressive disease during or after therapy, which was evaluated as any new lymph node lesion more than 1.5 cm in any axis or increase by 50% of previously involved sites from nadir detected by PET‐CT/CT or pathology confirmation according to the criteria proposed by Cheson et al.^14^ COX univariate and multivariate analysis were used to investigate the associations of MSI‐H and MSI‐L with PFS and OS, respectively. Kaplan–Meier analyses were used to construct PFS and OS curves. The log‐rank test was used to evaluate differences between groups.

The response to R‐CHOP/CHOP chemotherapy including complete response (CR), partial response (PR), stable disease (SD), and progressive disease (PD) which were evaluated by clinical evidence and PET‐CT or CT according to the response criteria for malignant lymphoma proposed by Cheson et al^14^ The correlations between CR after R‐CHOP/CHOP chemotherapy and the MSI phenotype were analyzed by univariate logistic regression analysis, and multivariate regression analysis was used to identify the independent predictors for chemotherapeutic response. Multivariate regression models were acquired by backward elimination in a model comprising the major predictive parameters, including age (≤60 vs >60 years), sex (male vs female), Ann Arbor stage (I‐II vs III‐IV), IPI scores (low(0‐2) vs high(3‐5)), B symptoms (no vs yes), serum LDH (≤240 vs >240), and molecular type (GCB vs non‐GCB). Data statistical analysis was carried out by IBM SPSS Statistics version 20.0 (IBM). *P* values less than .05 were recorded as statistically significant, and all tests were two‐sided.

## 3 RESULTS

### 3.1 Clinicopathologic characteristics of DLBCL

The clinicopathologic features of the 92 DLBCL cases are listed in Table 1. The mean patient age was 56 years (range, 18‐84 years). The molecular types of GCB and non‐GCB‐type were identified by the “IHC signature” of Hans et al.^15^ About 33 (35.9%) DLBCLs were diagnosed as GCB‐type DLBCLs, and 59 (64.1%) were non‐GCB DLBCLs. About 31 cases (33.7%) were nodal lymphomas, and 61 cases (66.3%) occurred in extranodal sites, including the gastrointestinal tract (38 cases), testes (19 cases), liver (1 case), kidney (1 case), ovary (1 case), and spleen (1 case). The mean Ki‐67 index was 76% (range, 30%‐95%). A total of 63 cases (68.5%) acquired CR after chemotherapy, and 29 cases (31.5%) did not achieve CR, which were recorded as noncomplete response (non‐CR), including 18 cases of PR and 11 cases of PD.

**Table 1:** Associations of MSI‐H and MSI‐L with the clinicopathologic characteristics of DLBCLs

Variables	Total case	MSI‐H (%)	MSS (%)	*P*‐value	Total case	MSI‐L (%)	MSS (%)	*P*‐value
Age								
≤60	51	3 (5.9)	48 (94.1)	.43	54	6 (11.1)	48 (88.9)	.98
>60	32	0 (0)	32 (100.0)		35	3 (8.6)	32 (91.4)	
Sex								
Male	59	2 (3.4)	57 (96.6)	1.00	62	5 (8.1)	57 (91.9)	.56
Female	24	1 (4.2)	23 (95.8)		27	4 (14.8)	23 (85.2)	
Primary site								
Nodal	30	0 (0)	30 (100.0)	.47	31	1 (3.2)	30 (96.8)	.23
Extranodal	53	3 (5.7)	50 (94.3)		58	8 (13.8)	50 (86.2)	
Ann Arbor Stage								
I‐II	60	3 (5.0)	57 (95.0)	.56	65	8 (12.3)	57 (87.7)	.46
III‐IV	23	0 (0)	23 (100.0)		24	1 (4.2)	23 (95.8)	
B Symptoms								
Yes	19	0 (0)	19 (100.0)	1.00	20	1 (5.0)	19 (95.0)	.66
No	64	3 (4.7)	61 (95.3)		69	8 (11.6)	61 (88.4)	
IPI scores								
Low (0‐2)	56	3 (5.4)	53 (94.6)	.55	59	6 (10.2)	53 (89.8)	1.00
High (3‐5)	27	0 (0)	27 (100.0)		30	3 (10.0)	27 (90.0)	
Serum LDH								
Normal (≤240)	57	3 (5.3)	54 (94.7)	.55	58	4 (6.9)	54 (93.1)	.31
High (>240)	26	0 (0)	26 (100.0)		31	5 (16.1)	26 (83.9)	
Type (IHC)								
GCB	29	0 (0)	29 (100.0)	.50	33	4 (12.1)	29 (87.9)	.91
Non‐GCB	54	3 (5.6)	51 (94.4)		56	5 (13.6)	51 (86.4)	
Therapy response								
CR	60	1 (1.7)	59 (98.3)	.18	62	3 (4.8)	59 (95.2)	.03*
Non‐CR	23	2 (8.7)	21 (91.3)		27	6 (22.2)	21 (77.8)	
Relapse or die in 2 y								
Yes	19	0 (0)	19 (100.0)	1.00	20	1 (5.0)	19 (95.0)	.66
No	64	3 (4.7)	61 (95.3)		69	8 (11.6)	61 (88.4)	

Abbreviations: CR, complete response; DLBCL, diffuse large B‐cell lymphoma; GCB, germinal center B cell; IHC, immunohistochemistry; IPI, International Prognostic Index; LDH, lactate dehydrogenase; MSI, microsatellite instability; Non‐CR, including partial response (PR), stable disease (SD) and progressive disease (PD).

* *P* values are significant at *P* < .05.

### 3.2 MSI and its associations with the clinicopathologic characteristics of DLBCLs

**Figure 2:**
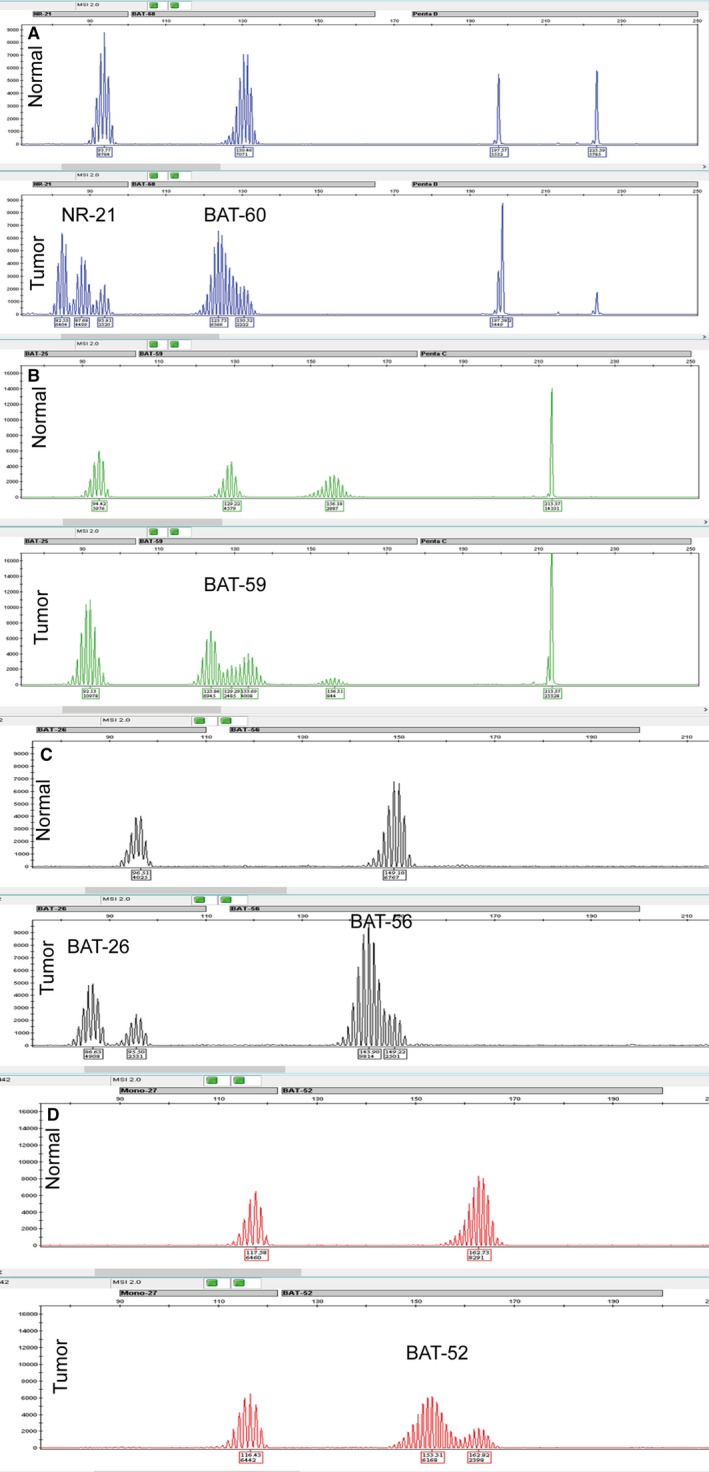
Microsatellite instability observed in diffuse large B‐cell lymphoma. The alterations of microsatellite markers including NR‐21, BAT‐60 (A), BAT‐59 (B), BAT‐26, BAT‐56 (C), and BAT‐52 (D) in a tumor sample with MSI‐H, compared with its normal control (the normal and tumor sample were indicated)

We detected the MSI phenotype by the Promega MSI Analysis System (version 2.0) in a panel of 92 DLBCL patients. Allelic profile alterations in microsatellite loci PCR products were found in 12 tumors and are shown in Table 2 and Figure 2. In 3 of the 12 tumors, microsatellite alterations were observed in at least 2 of the 8 markers, which were considered as MSI‐H based on the NCI criteria.^11^ The other nine tumors had alterations in one microsatellite marker, which was defined as MSI‐L. Therefore, the overall MSI frequency, including MSI‐H and MSI‐L, was 13.0% (12/92), the overall MSI‐H frequency was 3.3% (3/92), and 87.0% (80/92) were MSS in our cohort of DLBCLs. The clinicopathologic features of the 12 DLBCLs with MSI are listed in Table 3. We next investigated the correlations of MSI‐H and MSI‐L with the clinicopathologic parameters in DLBCLs, respectively. The MSI‐L phenotype was positively associated with non‐CR of R‐CHOP/CHOP chemotherapies (*P* = .03) (Table 1). However, there were no significant correlations of MSI‐H and MSI‐L with other clinicopathologic features, including patient age, sex, primary site, Ann Arbor stage, B symptoms, IPI scores, serum LDH level, and tumor molecular subtypes (Table 1).

**Table 2:** Microsatellite instability alterations of 12 cases of DLBCLs

Case No.	Microsatellite instability markers								Status
	NR‐21	BAT‐60	BAT‐25	BAT‐59	BAT‐26	BAT‐56	MONO‐27	BAT‐52	
1	N	P	N	N	N	P	N	N	MSI‐H
2	N	N	N	N	N	P	N	P	MSI‐H
3	P	P	N	P	P	P	N	P	MSI‐H
4	N	N	N	N	N	N	N	P	MSI‐L
5	N	N	N	N	N	N	N	P	MSI‐L
6	N	N	N	P	N	N	N	N	MSI‐L
7	N	N	N	P	N	N	N	N	MSI‐L
8	N	N	N	N	N	P	N	N	MSI‐L
9	N	N	N	N	N	N	N	P	MSI‐L
10	N	N	N	N	N	P	N	N	MSI‐L
11	N	N	N	N	N	P	N	N	MSI‐L
12	N	N	N	N	N	P	N	N	MSI‐L

Abbreviations: MSI‐H, high microsatellite instability; MSI‐L, low microsatellite instability; N, negative; P, positive.

**Table 3:** The clinicopathologic characteristics and the expression of MMR proteins of the 12 DLBCLs with MSI

Case No.	MSI	Gender	Type (IHC)	Age	PD	Primary site	B symptoms	Ki‐67 (%)	Ann Arbor Stage	High LDH (>240)	IPI scores	Chemotherapy	Response	MSH2	MSH6	MLH1	PMS2
1	MSI‐H	M	Non‐GCB	60	No	Testis	No	85	Ⅱ	No	1	R‐CHOP	CR	P	P	P	P
2	MSI‐H	F	Non‐GCB	47	No	Stomach	No	60	Ⅱ	No	0	CHOP	PR	P	P	P	P
3	MSI‐H	M	Non‐GCB	30	No	Colon	No	80	Ⅰ	No	0	CHOP	PR	N	N	P	P
4	MSI‐L	M	Non‐GCB	59	No	Testis	No	70	Ⅱ	Yes	1	CHOP	PR	P	P	P	P
5	MSI‐L	M	GCB	65	No	Stomach	No	60	Ⅱ	Yes	2	CHOP	PR	P	P	P	P
6	MSI‐L	M	Non‐GCB	76	No	Testis	No	60	Ⅰ	Yes	2	R‐CHOP	CR	P	P	P	P
7	MSI‐L	F	GCB	52	No	Ovary	No	70	Ⅱ	Yes	2	CHOP	PR	P	P	P	P
8	MSI‐L	F	Non‐GCB	49	No	Stomach	Yes	70	Ⅰ	Yes	0	CHOP	PR	P	P	P	P
9	MSI‐L	F	GCB	64	No	Breast	No	95	Ⅰ	Yes	0	R‐CHOP	CR	P	P	P	P
10	MSI‐L	F	Non‐GCB	55	No	Stomach	No	80	Ⅱ	Yes	0	CHOP	PR	P	P	P	P
11	MSI‐L	M	GCB	57	Yes	Retroperitoneal lymph node	No	90	Ⅲ	Yes	1	R‐CHOP	PD	P	P	P	P
12	MSI‐L	M	Non‐GCB	51	No	Small intestine	No	65	Ⅰ	Yes	1	R‐CHOP	CR	P	P	P	P

Abbreviations: CR, complete response; DLBCLs, diffuse large B‐cell lymphomas; F, female; GCB, germinal center B cell; IHC, immunohistochemistry; IPI, International Prognostic Index; LDH, lactate dehydrogenase; M, male; MMR, mismatch repair; MSI, microsatellite instability; MSI‐H, high microsatellite instability; MSI‐L, low microsatellite instability; N, negative nuclear expression; P, positive nuclear expression; PD, progressive disease; PR, partial response; R‐CHOP, rituximab, cyclophosphamide, doxorubicin, vincristine and prednisolone.

**Figure 3:**
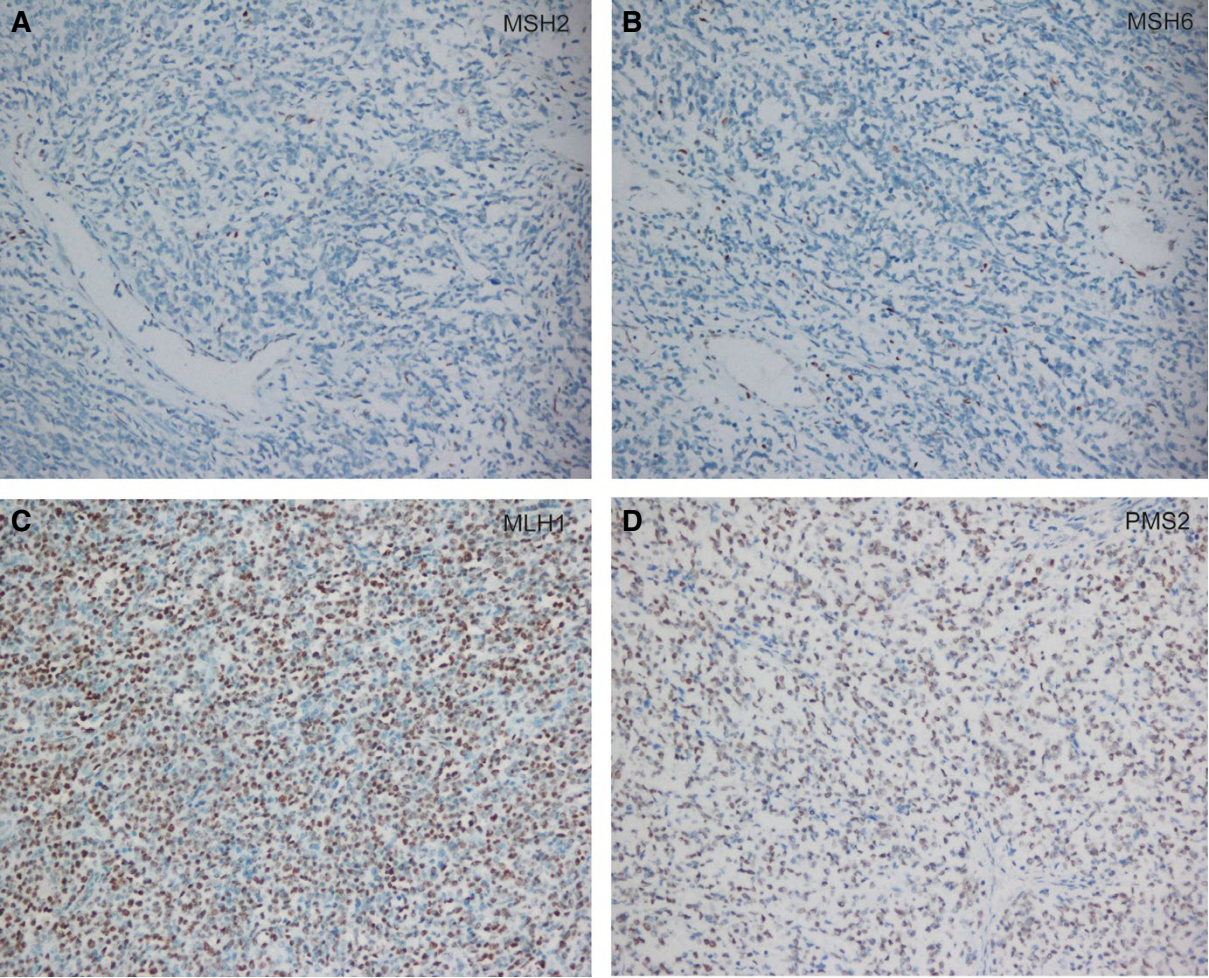
MSH2, MSH6, PMS2, and MLH1 immunohistochemistry in a diffuse large B‐cell lymphoma case with MSI‐H. A, B, MSH2 and MSH6 immunohistochemistry from a case with MSI‐H showed clonal loss of MSH2 (A) and MSH6 (B) in the tumor area with adjacent positive stromal cells acting as an internal control (×40 magnification); C, D, Positive MLH1 (C) and PMS2 (D) expression in >50% of the same tumor area and associated stromal cells (×10 magnification)

Microsatellite instability is widely considered as indicating dMMR of tumor cell. We next detected the protein expression of four major MMR genes, *MSH2*, *MSH6*, *MLH1*, and *PMS2*, in the 12 MSI tumors by immunohistochemistry (IHC). There was one MSI‐H case that exhibited a negative nuclear expression of *MSH2* and *MSH6* (Table 3, Figure 3), while the other two MSI‐H cases and nine MSI‐L cases had a positive nuclear expression of the four MMR proteins (Table 3).

### 3.3 Associations of MSI with prognosis and chemotherapeutic response in DLBCLs

**Figure 4:**
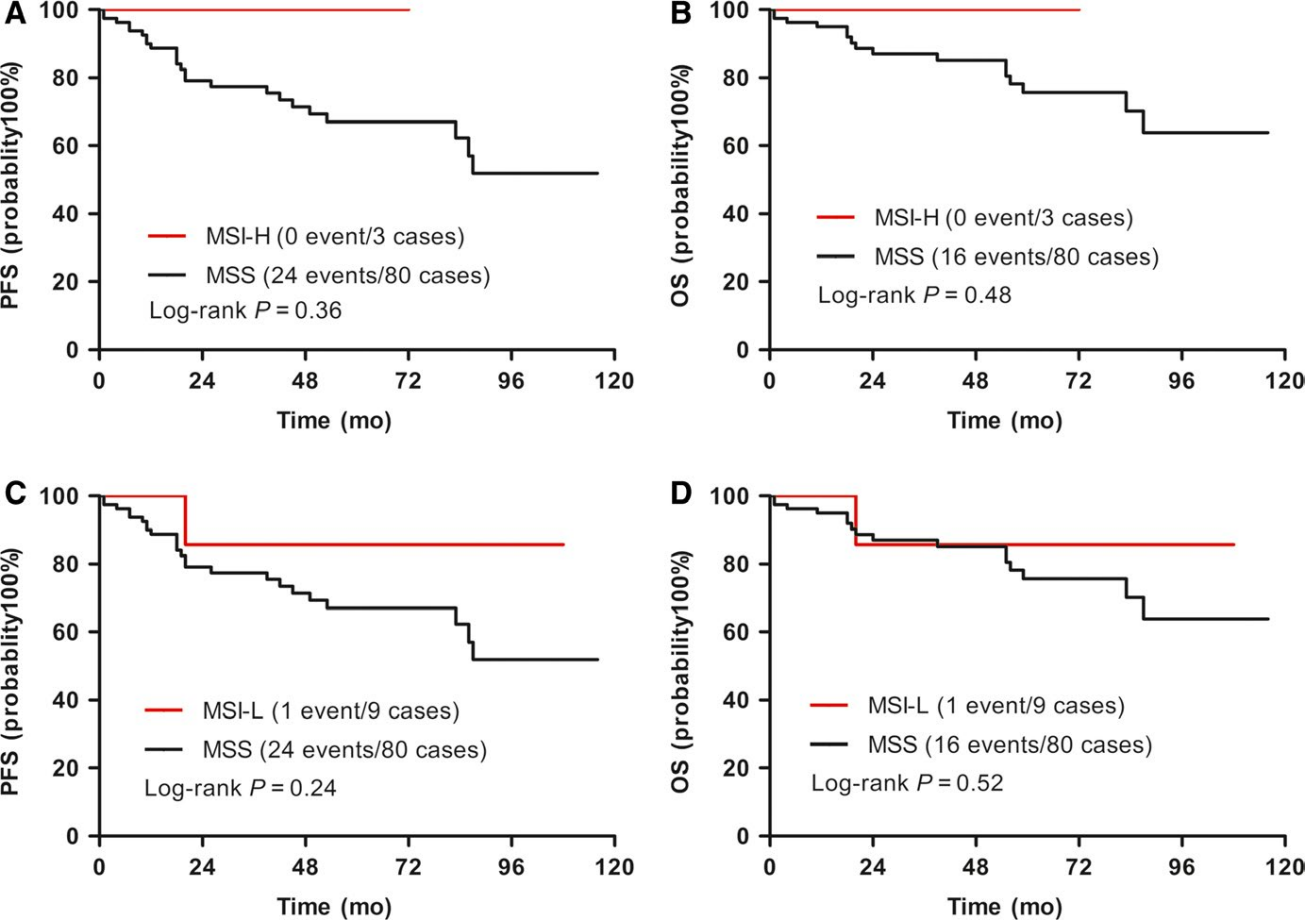
Kaplan–Meier curves for associations of MSI‐H and MSI‐L with progression‐free survival (PFS) and overall survival (OS) in diffuse large B‐cell lymphomas. A, B, MSI‐H was associated with favorable PFS and OS but not achieving statistical significance; C, D, MSI‐L was not significantly associated with PFS or OS (Log‐rank *P* values were shown)

The associations of MSI with prognosis were analyzed in 92 cases of DLBCL patients. Among 92 cases, there were 25 PFS events and 17 OS events. In the PFS events, 17 cases showed recurrence including 16 cases with new lymph node lesions diagnosed by FDG abnormal increase, CT or needle biopsy and one case recurred in previously involved sites diagnosed by excision biopsy. The MSI‐H and MSI‐L phenotype were not significantly correlated with PFS and OS in univariate and multivariate analysis (Table 4). Kaplan‐Meier analysis showed that there was a trend that MSI‐H patients had favorable PFS (*P* = .36) and OS (*P* = .48), but this trend did not have statistical significance (Figure 4). The MSI‐L phenotype was not significantly related with PFS (*P* = .24) and OS in DLBCLs (*P* = .52) (Figure 4).

**Table 4:** Univariate and multivariate analysis for associations of MSI‐H and MSI‐L with PFS and OS in DLBCLs

Variables	Univariate analysis			Multivariate analysis			Univariate analysis			Multivariate analysis		
	PFS			PFS			OS			OS		
	HR	95% CI	*P* value	HR	95% CI	*P* value	HR	95% CI	*P* value	HR	95% CI	*P* value
MSI												
MSS	1 [Reference]			1 [Reference]			1 [Reference]			1 [Reference]		
MSI‐L	0.32	0.043‐2.368	.265	0.379	0.048‐3.025	.360	.523	0.069‐3.951	.530	0.624	0.071‐5.458	.670
MSI‐H	0.0001	>0.0001	.980	0.0001	>0.0001	.981	0.0001	>0.0001	.984	0.0001	>0.0001	.986
Age												
≤60	1 [Reference]			1 [Reference]			1 [Reference]			1 [Reference]		
>60	2.699	1.220‐5.970	.014*	2.288	0.885‐5.914	.088	3.068	1.162‐8.099	.024*	2.494	0.792‐7.853	.118
Sex												
Male	1 [Reference]			1 [Reference]			1 [Reference]			1 [Reference]		
Female	1.280	0.573‐2.863	.547	1.021	0.423‐2.464	.964	0.757	0.265‐2.158	.602	0.701	0.226‐2.178	.539
Ann Arbor Stage												
I‐II	1 [Reference]			1 [Reference]			1 [Reference]			1 [Reference]		
III‐IV	2.385	1.040‐5.468	.040*	1.354	0.355‐5.172	.657	2.093	0.768‐5.703	.149	0.654	0.131‐3.256	.604
IPI scores												
Low (0‐1)	1 [Reference]			1 [Reference]			1 [Reference]			1 [Reference]		
High (2‐5)	2.715	1.186‐6.214	.018*	1.882	0.372‐9.534	.445	4.113	1.528‐11.07	.005*	3.843	0.601‐24.583	.155
B symptoms												
No	1 [Reference]			1 [Reference]			1 [Reference]			1 [Reference]		
Yes	0.980	0.367‐2.615	.968	0.817	0.280‐2.386	.711	0.799	0.229‐2.784	.725	0.569	0.141‐2.287	.427
Serum LDH												
≤240	1 [Reference]			1 [Reference]			1 [Reference]			1 [Reference]		
>240	0.932	0.388‐2.236	.874	0.775	0.224‐2.682	.688	1.455	0.535‐3.954	.463	1.113	0.286‐4.335	.877
Type(IHC)												
GCB	1 [Reference]			1 [Reference]			1 [Reference]			1 [Reference]		
Non‐GCB	1.226	0.527‐2.852	.636	0.890	0.352‐2.249	.805	1.102	0.406‐2.991	.849	0.772	0.256‐2.327	.645

Abbreviations: CI, confidence interval; DLBCL, diffuse large B‐cell lymphoma; GCB, germinal center B cell; HR, Hazard's ratio; IHC, immunohistochemistry; IPI, International Prognostic Index; LDH, lactate dehydrogenase; MSI‐H: Microsatellite instability‐high; MSI‐L: Microsatellite instability‐low; MSS: Microsatellite stable; OS: overall survival; PFS: progression‐free survival.

* *P* values are significant at *P* < .05.

The associations of MSI‐H and MSI‐L with the R‐CHOP/CHOP response were also investigated separately in our cohort. Among 92 cases, 63 cases acquired CR after chemotherapy, and 29 cases did not achieve CR, which were recorded as non‐CR, including 18 cases of PR and 11 cases of PD. Univariate logistic regression analysis showed that the MSI‐L phenotype was negatively associated with CR to R‐CHOP/CHOP chemotherapy in DLBCLs (odds ratio, 0.178; 95% CI, 0.041‐0.776; *P* = .022, Table 5). Multivariate regression analysis showed that the MSI‐L phenotype was an independent predictive factor for non‐CR to R‐CHOP/CHOP chemotherapy, irrespective of the clinicopathologic factors of DLBCLs (odds ratio, 0.144; 95% CI, 0.027‐0.761; *P* = .023, Table 5). However, there was no significant association between MSI‐H and CR in univariate and multivariate logistic regression analyses.

**Table 5:** Associations of MSI‐H and MSI‐L with CR of R‐CHOP/CHOP chemotherapy in DLBCLs

Variables	Univariate analysis			Multivariate analysis		
	OR	95% CI	*P*‐value	OR	95% CI	*P*‐value
MSI						
MSS	1 [Reference]			1 [Reference]		
MSI‐L	0.178	0.041‐0.776	.022*	0.144	0.027‐0.761	.023*
MSI‐H	0.178	0.015‐2.066	.168	0.261	0.015‐4.535	.357
Age						
≤60	1 [Reference]			1 [Reference]		
>60	1.85	0.614‐3.97	.027*	0.822	0.281‐2.411	.721
Sex						
Male	1 [Reference]			1 [Reference]		
Female	0.76	0.297‐1.947	.568	1.216	0.415‐3.565	.712
Ann Arbor Stage						
I‐II	1 [Reference]			1 [Reference]		
III‐IV	0.694	0.261‐1.845	.465	1.146	0.283‐4.637	.848
IPI scores						
Low (0‐1)	1 [Reference]			1 [Reference]		
High 2‐5)	0.567	0.226‐1.421	.226	0.897	0.176‐4.558	.895
B symptoms						
No	1 [Reference]			1 [Reference]		
Yes	0.817	0.287‐2.328	.705	0.865	0.265‐2.828	.811
Serum LDH						
≤240	1 [Reference]			1 [Reference]		
>240	0.318	0.126‐0.8	.015*	0.313	0.081‐1.216	.094
Type (IHC)						
GCB	1 [Reference]			1 [Reference]		
Non‐GCB	1.747	0.707‐4.313	.226	1.930	0.685‐5.444	.214

Abbreviations: CI, confidence interval; CR, complete response; DLBCL, diffuse large B‐cell lymphoma; GCB, germinal center B cell; IHC, immunohistochemistry; IPI, International Prognostic Index; LDH, lactate dehydrogenase; MSI‐H, Microsatellite instability‐high; MSI‐L, Microsatellite instability‐low; MSS, Microsatellite stable; OR, odd's ratio.

* *P* values are significant at *P* < .05.

## 4 DISCUSSION

Microsatellite instability reflects dMMR in tumor cells. Recent advances have shown that tumors with a high MSI status were correlated with a favorable response to PD‐1/PD‐L1 inhibitors and might also be associated with resistance to traditional chemotherapy. Identifying the MSI status in tumors might have important significance in clinical therapy and the prediction of patient outcomes. To date, few publications have reported the MSI status in DLBCLs based on a large sample size, and the associations of MSI status with clinicopathologic characteristics in DLBCLs have not been sufficiently investigated. Our study aimed to assess the MSI status in DLBCLs and to investigate the associations of MSI status with the clinicopathologic characteristics, chemotherapy response and prognosis in DLBCLs. In our study, 3 of the 92 (3/92, 3.3%) tumors exhibited MSI‐H, nine tumors (9/92, 9.8%) exhibited MSI‐L, and the overall MSI frequency was 13.1%. The MSI‐L phenotype was associated with poor response to chemotherapy and was an independent biomarker for predicting non‐CR to R‐CHOP/CHOP chemotherapy in our cohort of DLBCLs.

The MSI status in DLBCLs has been controversial, and to date, the data published in the literature lack unity. In addition, few groups have investigated the associations between MSI status and clinicopathologic features in DLBCLs with large samples. In this study, we performed MSI analysis using the Promega MSI Analysis System 2.0, which is a fluorescent PCR‐based assay that detects eight MSI markers. About 12 of 92 DLBCLs (13.1%) presented with MSI phenotypes, including three MSI‐H and nine MSI‐L tumors. There were no correlations between the MSI phenotype and clinicopathologic parameters, including patient age, sex, B symptoms, IPI scores, and Ann Arbor stage. The MSI frequency in our study appeared to be consistent with those reported in DLBCLs thus far. Miranda et al performed MSI analysis in 28 DLBCLs by detecting four microsatellite markers, and instability in one or two markers was examined in five DLBCL samples (5/28, 17.9%).^10^ Miyashita et al reported three MSI‐L tumors in 25 cases of DLBCLs (3/25, 12%).^16^ In line with our results, they demonstrated that there was no significant difference in the clinicopathologic variables of DLBCLs between tumors with MSI and those with MSS. They also found that MSI‐L was predominant in their cohort of non‐Hodgkin lymphomas, which was similar to our results. However, Couronne et al showed that no MSI was detected in 2 DLBCLs that separately exhibited a homozygous deletion of *MSH2*‐*MSH6* and a *PMS2* heterozygous deletion.^17^ Hiyama et al reported that MSI‐H was tested in 1 of 20 (5%) DLBCLs and MSI‐L was examined in 1 of 20 (5%) DLBCLs.^18^

Mismatch repair status could be detected by MSI analysis on tumor DNA and immunohistochemistry of the MMR proteins including MSH2, MSH6, MLH1, and PMS2 on tumor tissue. In colorectal cancers, tumors with MSI‐H often showed loss of expression of at least one of the four MMR proteins. However, recent studies have reported a rate of 3% to 10% of discordance between molecular MSI testing and MMR immunohistochemistry in colorectal cancers.^19^ In our study, one of the three MSI‐H cases showed negative expression of nuclear MSH2 and MSH6 proteins and other two cases showed intact nuclear expression of the four MMR proteins. The discordance between MSI PCR and immunohistochemistry might be caused by three reasons. First, some point mutations allowed normal MMR protein expression, but without retaining the MMR function. There were studies demonstrating that nontruncating and/or truncating mutations of MMR genes could lead to loss of function without absence of expression of MMR proteins, especially with MLH1.^20^ McCarthy et al suggested that somatic MSH6 variant (c.3261dupC; p.Phe1088Leufs*5) and two PMS2 missense variants (PMS2 c.1289C > T, p.Thr430Ile and PMS2 c.92T > C, p.Val31Ala) were present in the tumor area with retained expression of MSH6 and PMS2, which were MSI‐H.^21^ Second, other gene mutations except the four MMR gene mutations, such as MSH3, PMS1, and EPCAM could also result in loss of MMR function which caused MSI‐H. Chang et al demonstrated that germline mutations of AXIN2, POLE, and TGFBR2 also resulted in MSI‐H.^22^ Third, tumor heterogeneity might cause the discordance of MSI PCR and MMR proteins expression. Microsatellite instability was a heterogeneous event throughout the tumor in sporadic CRCs. Tachon et al reported a case that exhibited heterogeneous expression of MLH1 and PMS2 and had both MSI and MSS tumor areas.^23^ Therefore, multiple zone analyses, involving both MMR IHC and MSI detection, should be performed to better assess dMMR/MSI status. Finally, the sensitivity of MSI testing methods might cause false positive or false negative results. Analyzing a large panel of microsatellite markers and Next‐generation sequence (NGS) could improve MSI detection. Gustavo et al observed discordant results in eight cases and proposed the inclusion of HSP110 (T17) marker could confirm the MSI results.^24^ Nowak et al reported two colorectal carcinomas had dMMR predicted by NGS, but had intact MMR proteins expression by IHC. One carcinoma was MSS by PCR and the other was MSI‐H. Both carcinomas had a somatic POLE c.857C > G (p.Pro286Arg) mutation.^25^ Thus, NGS could improve the MSI detection sensitivity and simultaneously detected the pathogenic somatic mutations. The discordance of MSI PCR and MMR IHC in DLBCLs and the germline and somatic gene mutations involved in DLBCLs with MSI‐H needed more investigations in the future.

DNA mismatch repair activity regulates cellular sensitivity against different kinds of antitumor drugs, including 5‐FUand so on.^3^, ^4^ MSI is now considered as a crucial predictive biomarker to the tumor response against chemotherapy in colorectal cancer. In our study, we also investigated the associations of MSI status with chemotherapy response in DLBCLs. We found that in DLBCLs treated with R‐CHOP/CHOP therapies, the response to chemotherapy was significantly worse in tumors with MSI‐L (*P* = .022). The MSI‐L phenotype could be an independent predictor to non‐CR in DLBCLs (*P* = .023). MSI‐L might have a distinct underlying biology compared with MSI‐H and MSS tumors. In colorectal cancers, MSI‐L tumors might arise through the chromosomal instability carcinogenesis pathway, which caused by a series of genetic changes that involved the activation of proto‐oncogenes and inactivation of tumor‐suppressor genes. However, MSI‐H resulted from inactivation of MMR genes.^26^ Thus, MSI‐H and MSI‐L had distinct clinicopathologic features, prognosis and chemotherapy response. Amir et al suggested that MSI‐L was associated with advanced stage and inferior prognosis compared with MSI‐H and MSS in colorectal cancers.^27^ In DLBCLs, the underlying mechanisms of MSI‐H and MSI‐L needed further investigations.

In our current study, univariate and multivariate analysis showed that there was significant association between MSI‐L and the poor response of R‐CHOP/CHOP chemotherapy in DLBCLs. In addition, there was a trend that tumors with MSI‐H had a favorable PFS and OS but did not have statistical significance. One of the limitations of our study was the small cases of DLBCLs and the low portion of MSI cases. The results and the clinical validity of this biomarker should be confirmed in more cases and cohorts from multicenter.

Finally, our results might have valuable clinical significance. Our study demonstrated that MSI‐L could be an independent predictor to a noncomplete response of R‐CHOP/CHOP chemotherapy in DLBCLs, which might be used as an important biomarker in DLBCLs. Our findings indicated that MSI‐H and MSI‐L existed in DLBCLs, which implied that PD‐1/PD‐L1 inhibitors might be useful in DLBCLs and was helpful to expand patients who might obtain benefits from PD‐1/PD‐L1 inhibitors.

## 5 CONCLUSIONS

In conclusion, our study exhibited that 3 of the 92 (3/92, 3.3%) tumors exhibited MSI‐H and 9 tumors (9/92, 9.8%) exhibited MSI‐L, and the overall MSI frequency was 13.1%. The MSI phenotype was not correlated with the nuclear expression of four major MMR genes, *MSH2*, *MSH6*, *MLH1*, and *PMS2*. The MSI‐L phenotype was an independent predictive biomarker for the poor response of R‐CHOP/CHOP chemotherapy in DLBCLs. MSI‐H and MSI‐L was not significantly correlated with PFS and OS in DLBCLs. These findings suggested that the evaluation of MSI status had clinical significance in predicting the chemotherapy response in DLBCLs.

## CONFLICT OF INTEREST

All authors declare no conflict of interest.

## COMPLIANCE WITH ETHICAL STANDARDS

All procedures involving human participants were according to the ethical standards of Ethics Institutional Review Board (IRB) of Fudan University Shanghai Cancer Center and to the 1964 Helsinki declaration.

## Data Availability

The data generated during the present study are available from the corresponding author upon reasonable request.
